# A 2018 workshop: vaccine and drug ontology studies (VDOS 2018)

**DOI:** 10.1186/s12859-019-3191-9

**Published:** 2019-12-23

**Authors:** Junguk Hur, Cui Tao, Yongqun He

**Affiliations:** 1 Department of Biomedical Sciences, University of North Dakota School of Medicine and Health Sciences, Grand Forks, ND USA; 2 School of Biomedical Informatics, The University of Texas Health Science Center at Houston, Houston, TX USA; 3 Unit for Laboratory Animal Medicine, Department of Microbiology and Immunology, Center for Computational Medicine and Bioinformatics, University of Michigan Medical School, Ann Arbor, MI USA

**Keywords:** Ontology, Vaccine, Drug, VDOS workshop, Knowledge standardization, Data integration, Adverse event, Text mining

## Abstract

This Editorial first introduces the background of the vaccine and drug relations and how biomedical terminologies and ontologies have been used to support their studies. The history of the seven workshops, initially named VDOSME, and then named VDOS, is also summarized and introduced. Then the 7th International Workshop on Vaccine and Drug Ontology Studies (VDOS 2018), held on August 10th, 2018, Corvallis, Oregon, USA, is introduced in detail. These VDOS workshops have greatly supported the development, applications, and discussion of vaccine- and drug-related terminology and drug studies.

## Background

Drugs and vaccines are critical to public health worldwide. When we discuss drugs, we often mean chemical drugs. Vaccines are typically classified as biological drugs. Both follow similar paths and rules in terms of preclinical research, manufacturing, clinical trials, government approval, and post-licensure usage surveillance and monitoring. However, drugs and vaccines have many differences [1]. For example, vaccines are typically administered to healthy people, and drugs to patients with various diseases. As a result, it is more difficult to assess the safety of drugs compared to vaccines. The mechanisms are also different. Vaccines rely on the stimulation of protective immune responses, while drugs focus on pathway intervention. Drugs and vaccines are often regulated by different government agents. For example, in the USA, vaccines are regulated by the Center for Biologics (CBER) at the Food and Drug Administration (FDA), and drugs are regulated by the Center for Drug Evaluation and Research (CDER) at FDA. The safety surveillance of vaccines is mostly carried out by the Center for Disease Control (CDC), and the surveillance of drugs is managed by the FDA in the USA. Given these similarities and differences, it remains important for researchers and agents to collaborate and keep close communications for productive studies of both vaccines and drugs.

In the time of precision medicine and big data, there has been a huge challenge in organizing, integrating, and analyzing various vaccine and drug related data. The big data can be defined by the typical 4 V model: high volume, high variety, high velocity, and high veracity (data quality and data value) [2]. Such big data cannot be easily analyzed without machine interpretation and automated understanding and analysis. Such work will need machine-understandable standardized controlled terminologies. Furthermore, we also need to have machines to fully understand the meanings and relations of the controlled terms. This is why we need ontology, which is an extension to the early generation of biomedical terminologies.

Over the last decades, we have learned a lot about biomedical ontologies and terminologies and how they can support public health and basic biomedical research. Before the report of the Gene Ontology (GO) [3], we know many biomedical terminologies, such as MeSH (Medical Subject Headings) used in PubMed literature indexing, MedDRA for adverse event reporting, and SNOMED for electronic exchange of clinical health information. Ontology is an update of terminology. The wide use of GO made researchers understand the importance of ontology and motivate the development and applications of various ontologies. Nowadays, hundreds of ontologies, such as the Drug Ontology (DrON) [4] and the Ontology of Adverse Events (OAE) [5], are available. The more recent review article in *New England Journal of Medicine* highlights the critical role of ontologies in standardization, classification, integration, and analysis of various types of knowledge and data associated with diseases, mechanisms, and precision medicine [6].

The VDOS workshop series have continuously provided a platform for sharing new development and applications of vaccine- and drug-related ontologies, discussing challenges and solutions in the fields, and promoting collaborations among researchers. These workshops usually cover two main areas of topics. One topic is the ontology representation of drugs and vaccines and their associated topics such as adverse event, prescriptions, and molecular mechanisms. The other topic covers various applications of the ontologies in real-world situations such as text mining, machine learning, and software development. Basic and translational research as well as clinical subjects have been widely covered.

The 7th International Workshop on Vaccine and Drug Ontology Studies (VDOS-2018; https://sites.google.com/site/vdosworkshop/VDOS-2018) was held at Corvallis, Oregon, USA, on August 10, 2018. This workshop was part of the ninth International Conference on Biomedical Ontology (ICBO-2018). Overall, VDOS-2018 was another successful VDOS meeting. In this Editorial, we would like to first summarize the results of the previous VDOS meetings and then focus on the introduction of the papers presented in the VDOS-2018 workshop.

### Brief history of seven VDOSME/VDOS workshops

All the seven VDOS workshops so far were associated with the International Conference on Biomedical Ontology (ICBO) since 2012. The first workshop was named VDOSME-2012, standing for Vaccine and Drug Ontology in the Study of Mechanism and Effect 2012 [1]. VDOSME had an emphasis on mechanisms and effects of vaccines and drugs. To broaden our scope, we later changed our name to Vaccine and Drug Ontology Studies (VDOS). To simplify our discussion, we will simply refer to all the previous seven workshops as VDOS. These workshops were held for three times in USA, two times in Europe (Portugal and UK), and one time each in Austria and Canada (Table 1). Drs. Yongqun “Oliver” He and Cui Tao were co-organizers for all the meetings. Dr. Junguk Hur has served as a co-organizer for the last two VDOS workshops. In addition, Drs. Luca Toldo, Gully Burns, Darrell R Abernethy, Sivaram Arabandi, and Sirarat Sarntivijai had also served as VDOS co-organizers. Dr. Abernethy (1949–2017) [39], a former Associate Director for Drug Safety in the Office of Clinical Pharmacology at the US FDA, kindly accepted the invitation to join as a co-organizer for the VDOSME-2012 workshop and provided significant help and advice in the workshop organization and Editorial preparation [1]. Dr. Abernethy and all the co-organizers’ service and help are greatly appreciated.

**Table 1:** Summary of VDOS workshops since its inception

Name/Editorial	Venue	Time	Pull-length papers	Short papers	Keynote speakers
VDOSME-2012 [1]	Graz, Austria	7/21/2012	6 [7–12]	3	
VDOS-2013 [13]	Montreal, Canada	7/72013	6 [4, 14–18]	3	
VDOS-2014 [19]	Houston, USA	10/7/2014	5 [20–24]	1	1
VDOS-2015	Lisbon, Portugal	7/27/2015	5 [25–29]	1	
VDOS-2016	Corvallis, OR, USA	8/1/2016	7* [30–34]		
VDOS-2017	Newcastle-upon-Tyne, UK	9/13/2017	4 [35–38]		
VDOS-2018 (Note: current Editorial)	Corvallis, OR, USA	8/10/2018	4 (Note: current papers)	1	
Total			37–35**	9	1

* Out of seven, five papers were published in the journal. ** Out of 37, 35 papers were published in peer-reviewed journals

In total, these workshops have accepted 37 full-length papers and 9 short papers (Table 1). We used EasyChair (http://easychair.org) to manage our paper submissions and reviewing. All of the papers were peer-reviewed by at least two experts before their acceptance. All of them were orally presented in the workshops and the authors were invited for submitting an extended research article for publication in peer-reviewed journals. Overall, all of these papers except two (Table 1) have been published in peer-reviewed journals, including *Journal of Biomedical Semantics*, *Biomedical Informatics Insights*, or *BMC Bioinformatics*.

We have had one keynote speaker, Dr. Khalid F. Almoosa, School of Biomedical Informatics, University of Texas Health Science Center at Houston, USA, to present in VDOS-2014 held at Houston. Dr. Almoosa was invited as an expert in the field of clinical bioinformatics research. The invitation of only one keynote presentation is largely due to the shortage of funding. It would be ideal to obtain additional funding to invite experts and governmental officials to present relevant topics.

### VDOS-2018 workshop presentation report

In the VDOS 2018 workshop, various international attendees, including paper presenters, senior academic and government scientists, postdoctoral fellows, and graduate students, participated in this workshop. This year, four full-length papers and one short-length paper were accepted for oral presentations at the workshop after a peer-review process with each submission reviewed by at least three independent reviewers. After one additional round of independent peer reviewing on their extended version, with the reviewers’ comments taken care of, by the workshop co-organizers and the journal editors, four full-length papers [40–43] have been accepted for publication in the current thematic issue of the BMC Bioinformatics.

In the area of ontology mapping, **Bona et al.** [40] proposed to improve Drug Ontology (DrOn) [44], a modular extensible ontology of drug products, their ingredients, and their biological activity. DrOn was originally created to enable comparative effectiveness and health services researchers to query National Drug Codes (NDC), a 10-digit 3-segment numbering system to uniquely represent drug products, issued by the U.S. Food and Drug Administration (FDA). DrOn is constructed based on the RxNorm [45] drug terminology and Chemical Entities of Biological Interest (ChEBI) [46]. This paper presented an enhancement of the DrOn with semantically rich representations of NDC, which resulted in a prototype that demonstrates the feasibility of this approach. A full accounting of NDC and RxNorm unique concept identifiers as information content entities and of the processes involved in managing their creation and changes has been implemented. The modeling efforts have considered not only assigning a NDC code but also deactivating a NDC, through using some best practice and tooling available from the OBO foundry community. Enabling the correct mappings between NDC codes and RxNorm codes in the context of the DrON ontology is a significantly meaningful effort. The enhanced DrOn will be especially useful in determining what packaged drug product an occurrence of an NDC in a database denotes in case the same code is assigned to different products at different points in time.

In the area of ontology development and representation, **Ong et al.** [41] developed a Vaccine Investigation Ontology (VIO) as an extension of the Vaccine Ontology (VO) and applied VIO to classify the different experimental variables and relations among them in the vaccine research. Different responses in the host to the same vaccine are frequently observed in vaccine studies; therefore, it systematically represents different experimental and analysis conditions. The development of VIO followed the eXtensible Ontology Development (XOD) principles [47] and is aligned with the Basic Formal Ontology (BFO) principles [48]. As a use case, the authors reanalyzed two microarray datasets of live attenuated Yellow Fever vaccine YF-17D, by Gaucher et al. 2008 [49] and Querec et al. [50], and reanalyzed differentially expressed genes (DEGs) affected by vaccination and their enriched biological functions in terms of Gene Ontology (GO) and Reactome pathways using the same approach as given in these publications. Surprisingly, the study showed a quite significant difference in terms of the number and constitution of differentially expressed genes compared to the published results, which were likely due to the software package differences. Significant differences in DEGs between two studies were also noted, while the GO enrichment results had more overlapping than the gene lists and the enriched pathway lists. This ontology-based analysis framework using VIO will be useful in representing heterogeneous data of host responses to vaccines, where differences in specific variables might explain different results drawn from similar studies.

**Amith et al.** [42] presented the development of Patient Health Information Dialogue Ontology (PHIDO) to model dialogue interaction related to health information. PHIDO is the result of an early simulation study involving a conversational agent discussing human papillomavirus (HPV) vaccine information to patients and fielding their questions over the course of the counseling session [51]. From the collected dialogue exchanges between the user and the simulated agent, the researchers derived the conceptual level that describes four basic class abstractions - Discussion, Goal, Speech Task, and Utterance. From this, the ontology offers a foundational framework that could permit the construction of dialogue interaction for software agents. This article outlines several dialogue interaction patterns using the ontology’s features, and introduces a general algorithm for a software engine to harnesses the PHIDO. With PHIDO, there is the potential to formalize health dialogue interaction between software agents and users, align the dialogue ontology to health behavioral models, and provide machines the interaction intelligence to converse in a clinical environment. The overall outcome of this work is to advance the research in using conversational agents in counseling situations for healthcare, particularly in vaccine counseling, which has a dramatic effect on improving vaccination uptake. This is one of few attempts to use ontologically modeling for dialogue systems. The researchers’ future goals include the development of a software engine that utilizes PHIDO to counsel users on the HPV vaccine.

Lastly, **Tiftikci et al.** [43] presented a machine learning (ML)- and rule-based system for identifying adverse drug reaction (ADR) mentions in the text of drug labels and their normalization through the Medical Dictionary for Regulatory Activities (MedDRA) dictionary. ADRs, unwanted or unexpected events from using drugs, are a major safety concern, and drug labels describe established ADRs for the given drug. Systematically identifying ADRs from drug labels is critical in multiple aspects, allowing a comparison of ADRs from different manufacturers for the same active ingredient and enabling post-marketing safety analysis by identifying new ADRs not presented in the labels. This paper challenged the ADR identification task, as part of the Text Analysis Conference (TAC) Adverse Drug Reaction 2017 challenge (https://tac.nist.gov/2017/), using ML- and rule-based approaches. The ML approach employed a deep learning architecture, integrating bi-directional Long Short-Term Memory (Bi-LSTM), Convolutional Neural Network (CNN), and Conditional Random Fields (CRF) for entity recognition. Rule- and dictionary-based approach was implemented on their in-house text mining system, SciMiner [35, 52], which was also used for normalizing the identified ADR mentions to MedDRA terms. The ML-based approach outperformed the rule-based approach, achieving a 77.0% F1 score on the task of ADR mention recognition and 82.6% micro-averaged F1 score on the task of ADR normalization. This paper was the first study of utilizing ML approaches presented in VDOS workshops, and we expect to see more studies utilizing similar approaches in the future VDOS workshops.

## Discussion

Overall, the VDOS-2018 workshop covered six full-length paper representations and offered a platform for sharing the results of vaccine- and drug-related ontology development and applications. A lot of positive feedbacks were provided. We also expect to continue this workshop series in the future and make it an attractive event for more and more ontology and application developers and users.

## Data Availability

Not applicable.

## Abbreviations

ADR: Adverse drug reaction
BFO: Basic Formal Ontology
Bi-LSTM: Bi-directional Long Short-Term Memory
ChEBI: Chemical Entities of Biological Interest
CNN: Convolutional Neural Network
DEGs: Differentially expressed genes
DrOn: Drug Ontology
GO: Gene Ontology
HPV: Human papillomavirus
MedDRA: Medical Dictionary for Regulatory Activities
ML: Machine learning
NDC: National Drug Codes
PHIDO: Patient Health Information Dialogue Ontology
TAC: Text Analysis Conference
VDOS: Vaccine and drug ontology studies
VIO: Vaccine Investigation Ontology
VO: Vaccine Ontology
XOD: eXtensible Ontology Development
